# Comparison of Three Screening Test Kits for G6PD Enzyme Deficiency: Implications for Its Use in the Radical Cure of Vivax Malaria in Remote and Resource-Poor Areas in the Philippines

**DOI:** 10.1371/journal.pone.0148172

**Published:** 2016-02-05

**Authors:** Fe Esperanza Espino, Jo-Anne Bibit, Johanna Beulah Sornillo, Alvin Tan, Lorenz von Seidlein, Benedikt Ley

**Affiliations:** 1 Department of Parasitology, Research Institute for Tropical Medicine, Department of Health, Alabang, Muntinlupa City, Philippines; 2 Department of Epidemiology and Biostatistics, Research Institute for Tropical Medicine, Department of Health, Alabang, Muntinlupa City, Philippines; 3 Mahidol Oxford Research Unit, Bangkok, Thailand; 4 Menzies School of Health Research and Charles Darwin University, Global and Tropical Health Division, Darwin, Australia; Agency for Science, Technology and Research—Singapore Immunology Network, SINGAPORE

## Abstract

**Objective:**

We evaluated a battery of Glucose-6-Phosphate Dehydrogenase diagnostic point-of-care tests (PoC) to assess the most suitable product in terms of performance and operational characteristics for remote areas.

**Methods:**

Samples were collected in Puerto Princesa City, Palawan, Philippines and tested for G6PD deficiency with a fluorescent spot test (FST; Procedure 203, Trinity Biotech, Ireland), the semiquantitative WST8/1-methoxy PMS (WST; Dojindo, Japan) and the Carestart G6PD Rapid Diagnostic Test (CSG; AccessBio, USA). Results were compared to spectrophotometry (Procedure 345, Trinity Biotech, Ireland). Sensitivity and specificity were calculated for each test with cut-off activities of 10%, 20%, 30% and 60% of the adjusted male median.

**Results:**

The adjusted male median was 270.5 IU/10^12^ RBC. FST and WST were tested on 621 capillary blood samples, the CSG was tested on venous and capillary blood on 302 samples. At 30% G6PD activity, sensitivity for the FST was between 87.7% (95%CI: 76.8% to 93.9%) and 96.5% (95%CI: 87.9% to 99.5%) depending on definition of intermediate results; the WST was 84.2% (95%CI: 72.1% to 92.5%); and the CSG was between 68.8% (95%CI: 41.3% to 89.0%) and 93.8% (95%CI: 69.8% to 99.8%) when the test was performed on capillary or venous blood respectively. Sensitivity of FST and CSG (tested with venous blood) were comparable (p>0.05). The analysis of venous blood samples by the CSG yielded significantly higher results than FST and CSG performed on capillary blood (p<0.05). Sensitivity of the CSG varied depending on source of blood used (p<0.05).

**Conclusion:**

The operational characteristics of the CSG were superior to all other test formats. Performance and operational characteristics of the CSG performed on venous blood suggest the test to be a good alternative to the FST.

## Introduction

Glucose-6-phosphate dehydrogenase deficiency (G6PDd) is among the most common enzymopathies and affects at least 400 million individuals worldwide [1–4]. More than 185 different variants of G6PDd have been reported with a spectrum of associated enzyme deficiencies [1, 5]. There is a strong overlap between areas of high G6PDd and vivax malaria endemic areas [6, 7], presumably the result of natural selection due to some degree of protection from malaria infection [8].

While G6PDd offers some protection against malaria, it complicates eradication of the dormant hypnozoite stage of *Plasmodium vivax* from the human host (radical cure). The only class of drugs currently on the market to eliminate hypnozoites, 8-aminoquinolines, can trigger severe hemolysis in G6PDd individuals [9]. There are a number of 8-aminoquinolines under development, among which tafenoquine is the most promising [10, 11], while primaquine is the only currently licensed 8-aminoquinoline. The risk of primaquine-induced hemolysis in G6PD deficient individuals is a significant public health concern in malaria endemic countries and a major barrier for better vivax malaria control[12].

There are several qualitative screening methods on the market for the detection of G6PD deficiency [13, 14]. The majority, if not all qualitative tests, are based on estimating enzyme activity by visualizing the reduction of NADP^+^ to NADPH, either directly or indirectly through secondary reactions. The most widely used qualitative test is the fluorescent blood spot test [15], a test format that requires a water bath and an ultraviolet (UV) light. In contrast, the recently developed Carestart G6PD Rapid Diagnostic Test (RDT) (CSG; Access Bio, New Jersey, USA) [16] is a rapid point-of-care test. The design follows the cassette format of most malaria RDTs and is based on the reduction of colourless nitro blue tetrazolium dye to dark colour formazan [16]. A comparable principle applies to the G6PD WST 8/1 PMS Methoxy Kit (Dojindo Co., Japan) [17].

The Philippines’ Malaria Program targets malaria elimination by 2030. For the past 30 years, it has routinely administered primaquine for radical cure of vivax malaria without testing for G6PD deficiency. In 2012, the Program documented an 18.3% recurrence of parasitemia within six months after 15 mg daily for 14 days primaquine administration among 93 vivax malaria patients (unpublished data). In consequence, a 30 mg primaquine dose is recommended in the 2015 Philippines’ Malaria Program Manual of Operations (Baquilod, M., Department of Health, Philippines, personal communication). Recently revised World Health Organization (WHO) recommendations suggest to test for G6PD deficiency before radical cure of vivax malaria [18]. A robust and reliable point-of-care assay with convenient operational characteristics would be highly desirable for deployment in remote and resource-poor areas in the Philippines where malaria diagnosis and treatment are carried out by community health workers. The aim of this study was to evaluate three point-of-care tests for their applicability in this situation.

## Materials and Methods

### Study Site and population

Samples were collected as part of a cross-sectional survey of G6PD deficiency among high school students in Puerto Princesa City, Palawan, Philippines. The island province Palawan is located in the west of the country and is divided into 367 political subunits, barangays. Palawan reports the highest annual malaria incidence of the country. In 2013, 4,662 of 7,449 (62.6%) of the malaria cases were reported from the province, where 15.3% of all reported infections from the area were due to *P*. *vivax* (Department of Health, personal communication).

### Study Population and Sample Collection

Ten schools were randomly selected from a list of all high schools in Puerto Princesa. All students attending one of the selected schools were screened for eligibility. A randomly selected subset of all students aged 12 years and above who did not suffer from acute or chronic illnesses were invited to participate. After the participants gave written assent and their parents/legal guardians provided written informed consent, information on sex and age was collected. Two hundred fifty microliters of capillary blood and 3 mL of venous blood were collected in an EDTA microtainer and EDTA vacutainer, respectively. All samples were stored at 4–8°C. Capillary blood was processed within 48 hours of collection while 73% of the venous blood samples were analyzed within two days after collection and the rest in 3 to 6 days. The stability of the samples was not tested over six days of storage.

### Sample Testing

The quantitative G6PD activity of each sample was assessed using the Trinity Biotech quantitative assay, Procedure No. 345 (Trinity Biotech, Ireland) adapted on a Mindray BA-88A (Mindray, China) spectrophotometer following manufacturer’s instructions. Three different controls from Trinity Biotech (deficient G5888, intermediate G5029 and normal G6888) were used for every run. Runs were considered valid if control values fell within a given range provided by the manufacturer. G6PD activities were expressed as international units/red blood cells (IU/10^12^ RBC). Red blood cell count of each venous EDTA sample was performed on a Sysmex XS 800i (Sysmex, Japan).

Three qualitative test assays were evaluated according to manufacturers’ recommendation. The fluorescent blood spot method (FST; Procedure No. 203—Trinity Biotech, Ireland) was performed using 10 μL of capillary blood. Briefly, the blood sample was mixed with 200 μL substrate solution, and a drop of the blood-substrate mixture was then transferred to a filter paper. The tube with the remaining blood-substrate mixture was incubated at 37°C. A second and third drop of blood-substrate mixture were transferred to the filter paper, 5 and 10 minutes after incubation. All filter papers were dried before the blood spots were read under long-wave UV light at a wavelength of 365 nm. A sample was considered as G6PD normal if the corresponding blood spot showed moderate to strong fluorescence at 5 minutes, and strong fluorescence at 10 minutes incubation. The sample was considered to have intermediate G6PD activity if the blood spot showed weak fluorescence at 5 minutes and moderate fluorescence at 10 minutes. The result was considered G6PD deficient if only very faint or no fluorescence was observed after 10 minutes incubation. Controls for normal, intermediate and deficient blood spots were made for each batch of test using Trinity Biotech controls.

The visual colorimetric method (G6PD Assay Kit WST—Dojindo Co., Japan) was performed on capillary blood, according to package insert. A mixture of 760 μL water, 20 μL substrate mixture and 20 μL dye were added to a 1.5 mL microtube to prepare the assay solution. A total of 5 μL of capillary blood was added and the content of the microtube was mixed well and incubated at 37°C for 30 minutes. After incubation, 10 μL hydrochloric acid was added to stop the reaction. The resulting color intensity of the sample solution was compared with color intensities of normal and deficient controls (Trinity Biotech normal G6888 and deficient G5888) prepared in parallel.

A rapid chromatographic test, Carestart™ G6PD Screening Kit (CSG; Access Bio, USA), was performed on a subset of samples. Testing was repeated on capillary and venous blood. In either case, 2 μL of blood sample was added to the sample well of the test cassette, followed by 2 drops of assay buffer. Test results were interpreted as normal when a distinct purple color appeared in the reading window within 10 minutes. No color change or a very faint purple color was classified as deficient.

### Assessment of Operational Features

Information on operational characteristics was based on the package insert and instructions provided by the manufacturers in 2014. The prices of FST and WST were based on the cost of the assay in US dollars within the Philippines in 2014 and the price of CSG was based on a paper by Roca-Feltrer et al [19].

### Data Management and Analysis

All case record forms were collected, checked for completeness and double entered into a database using EpiInfo/EpiData version 3.5.1. (CDC, USA). Statistical analysis was done using STATA version 13.0. (StataCorp, USA). For visualization of results, the dot plot maker from Vanderbilt University (http://data.vanderbilt.edu/~graywh/dotplot/, last accessed on 17 September 2015) was used.

Spectrophotometry (Trinity Biotech quantitative assay, Procedure No. 345 –Trinity Biotech, Ireland) was considered as the reference method. G6PD deficiency was defined as described earlier [13] and calculated per 10^12^ RBC and per gHb. In the absence of a standardized G6PD threshold activity, we calculated the median G6PD activity as measured by spectrophotometry of all male students and excluded all results with ≤10% G6PD activity of the derived median. We re-calculated the median based on the remaining samples of male participants and defined the resulting G6PD activity as 100%. Based on this reference value, G6PD cut-off activities for G6PD deficiency at 10%, 20%, 30% and 60% were calculated [13]. As hemoglobin content per dL blood and number of red blood cells do not match perfectly, the denominator for G6PD activity varies slightly. We considered the number of RBCs to be the more precise measure; test performance, therefore, was calculated based on the G6PD activity in IU/10^12^ RBC.

The FST results were defined in two ways: 1) FSTdefint—FST deficient and intermediate test results were defined as G6PDd, and 2) FSTdef—only FST deficient results are defined as G6PDd and intermediate results as G6PD normal. The CSG was performed twice, each on capillary (CSGcap) and venous (CSGven) sample for comparison. G6PDd prevalence within the population was calculated per cut-off activity as measured by spectrophotometry and presented as percentage of the study population with activities at or below the respective cut-off activity.

For the purpose of analysis, a positive test result was defined as a test result that indicates G6PD deficiency. Sensitivity and specificity were calculated using a standard formula [13]. In SE Asia, point-of-care test will primarily serve to guide 8-aminoquinoline treatment of vivax malaria, sensitivities of all tests were compared at 30% G6PD activity [20]. A lower cut-off may apply for guidance of single low-dose primaquine therapy to prevent the transmission of falciparum malaria. Differences in proportions were calculated using Chi-square and Fisher’s exact test for the results of FST and WST, as appropriate. McNemar’s test was used for comparing differences in proportion for the CSG. Confidence intervals were calculated using the exact method.

### Ethics

Ethical approval (Study No. 2010–046) for the study was granted by the Institutional Review Board of the Research Institute for Tropical Medicine, Department of Health, Philippines, and was conducted according to the principles expressed in the Declaration of Helsinki. Permission was also obtained from the Department of Education, Division of Puerto Princesa City, Philippines.

## Results

A total of 621 samples were collected between December 2011 and November 2012. The ratio of men to female was 1:1.75, and the median age was 15 years (range: 12 to 26 years of age).

### Quantitative Test Results

Spectrophotometry was performed on all 621 samples. The adjusted male median [13] was defined as 100% G6PD activity and was calculated at 270.5 IU/10^12^ RBC (iqr: 227.5–317.0 IU/10^12^ RBC) from 208/227 samples or 9.8 IU/g Hb (iqr: 8.4–11.28 IU/g Hb) from 204/227 samples. For comparison, the adjusted female median was 259 IU/10^12^ RBC (iqr: 203.0–307.5 IU/ 10^12^RBC) calculated from 383/394 samples.

**Fig 1:**
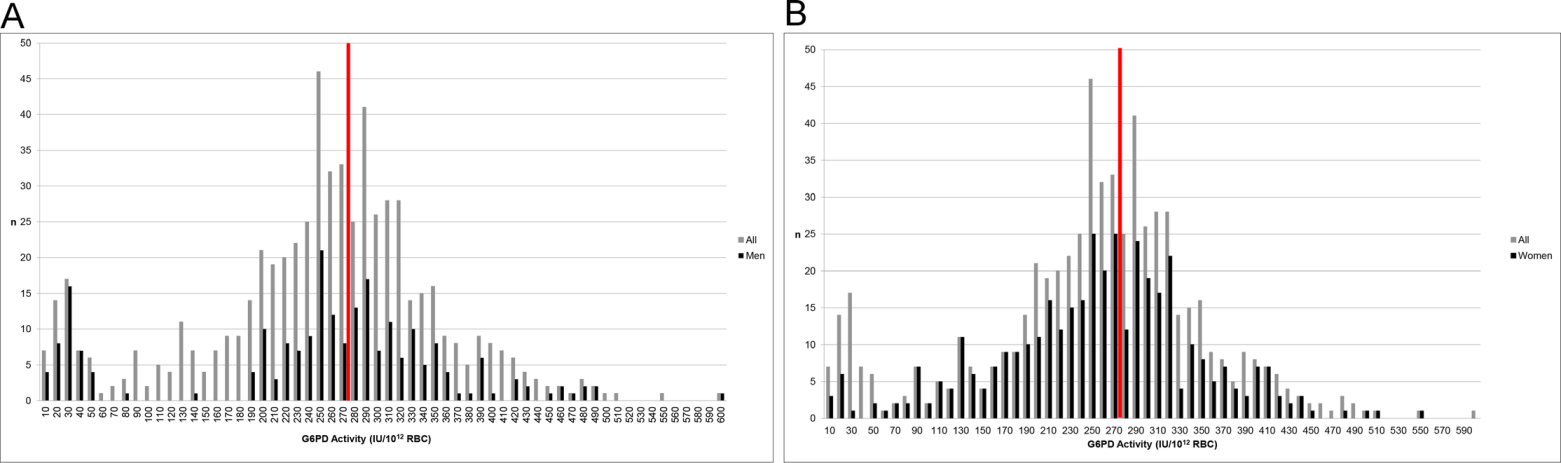
Distribution of G6PD activity among the study population. (A) G6PD activity among male students. (B) G6PD activity among female students. Legend: Red line indicates 100% G6PD activity (the adjusted male median)

Cut-off activities at 10%, 20%, 30% and 60% of the adjusted male median were calculated per 10^12^ RBC and per gHb) (Table 1). Proportions varied slightly; however, they did not differ significantly (all P>0.05). Based on spectrophotometry, there were significantly more male than female students (17.6% versus 4.3%, respectively; p<0.01) with G6PD activities below ≤30% of the adjusted male median G6PD activity (Table 1 and, Fig 1A and 1B).

**Table 1:** Proportion of participants by sex and G6PD activity.

	Percent G6PD cut-off activity (in IU/10^12^ RBC / IU/g Hb)					
	≤10 (≤27.05 / ≤0.98)	≤20 (≤54.1 / ≤1.96)	≤30 (≤81.15 / ≤2.94)	≤60 (≤162.3 / ≤5.88)	≤100 (≤270.5 / ≤9.80)	>100 (>270.5 / >9.80)
% Female (n = 394)	2.5 / 2.5	3.3 / 3.3	4.3 / 4.6	16.8 / 16.8	58.6 / 57.1	41.4 / 42.4
**% Male (n = 227)**	11.5 / 10.1	17.2 / 16.7	17.6 / 17.2	18.1 / 17.6	54.2 / 54.6	45.8 / 44.1
**% All (n = 621)**	5.8 / 5.3	8.4 / 8.2	9.2 / 9.2	17.2 / 17.1	56.9 / 56.2	42.9 / 43.0

### Qualitative Test Results

The fluorescent blood spot test (FST; Procedure No. 203, Trinity Biotech) and the WST 8/1 PMS-Methoxy (WST; Dojindo, Japan) were each performed on 621 samples. The Carestart G6PD RDT (CSG; Carestart; Accessbio, USA) was performed on a subset of 302 of the same samples. Performance of all test results at different cut-off activities is presented in Table 2. When comparing sensitivities at a threshold activity of 30%, the FSTdefint was significantly more sensitive than the CSGcap (p = 0.001) and the WST (p = 0.026); however, performance was comparable to the CSGven (p = 0.625). Sensitivity of the CSGven was significantly higher than sensitivity of CSGcap (p = 0.041).

**Table 2:** Performance of qualitative test assays at different cut-off activities (males and females).

	Sensitivity % (95% CI)				Specificity (95% CI)			
G6PD cut off activity in %	10	20	30	60	10	20	30	60
**Fluorescent Spot Test (FSTdefint)**^a^	97.2 (85.5–99.9)	96.2 (86.8–99.5)	96.5 (87.9–99.5)	67.3 (57.5–76.0)	91.9 (89.5–94.0)	94.4 (92.2–96.1)	95.2 (93.1–96.8)	98.1 (96.5–99.1)
**Fluorescent Spot Test (FSTdef)**^a^	91.7 (78.2–97.1)	88.5 (77.0–94.6)	87.7 (76.8–93.9)	47.7 (38.4–57.0)	96.4 (94.6–97.6)	98.6 (97.3–99.3)	99.3 (98.2–99.7)	99.4 (98.3–99.8)
**Colorimetric Test (WST)**^a^	88.9 (73.9–96.9)	86.5 (74.2–94.4)	84.2 (72.1–92.5)	48.6 (38.8–58.5)	95.6 (93.6–97.1)	97.7 (96.1–98.8)	98.2 (96.8–99.1)	98.8 (97.5–99.6)
**CSG (capillary blood sample)**^b^	63.6 (30.8–89.1)	71.4 (41.9–91.6)	68.8 (41.3–89.0)	47.1 (29.8–64.9)	91.4 (87.6–94.4)	92.4(88.7–95.2)	92.7 (89.0–95.4)	94.0 (90.5–96.5)
**CSG (venous blood sample)**^b^	90.9 (58.7–99.8)	92.9 (61.0–99.8)	93.8 (69.8–99.8)	64.7 (46.5–80.3)	93.5 (90.0–96.0)	94.4 (91.1–96.8)	95.1 (91.9–97.3)	97.4 (94.7–98.9)

^a^n = 621

^b^n = 302.

Heterozygote females may trigger a G6PD normal test result in qualitative assays, despite having very low G6PD activities when measured by spectrophotometry. Table 3 presents the performance of the qualitative test when based on samples from male participants only (n = 227). When comparing sensitivities at 30% G6PD cut-off activity based on samples from the entire study population (Table 2) to men only (Table 3), no significant difference was observed (all p>0.05).

**Table 3:** Performance of qualitative test assays at different cut-off activities (males only).

	Sensitivity % (95% CI)				Specificity (95% CI)			
G6PD cut off activity in %	10	20	30	60	10	20	30	60
**Fluorescent spot Test (FSTdefint)**^a^	96.2 (80.3–99.9)	94.9 (82.7–99.4)	95.0 (83.1–99.4)	92.7 (80.1–98.5)	93.0 (88.6–96.1)	98.9 (96.2–99.9)	99.5 (97.0–100.0)	99.5 (97.0–100.0)
**Fluorescent Spot Test (FSTdef)**^a^	96.2 (80.4–99.9)	94.9 (82.7–99.4)	95.0 (83.1–99.4)	92.7 (80.1–98.5)	93.5 (89.2–96.5)	99.5 (97.1–99.9)	100.0 (98.1–100.0)	100.0 (98.1–100.0)
**Colorimetric Test (WST)**^a^	84.6 (65.1–95.6)	84.6 (69.5–94.1)	82.5 (67.2–92.7)	80.5 (65.1–91.2)	94.0 (89.8–96.9)	99.5 (97.1–100.0)	99.5 (97.1–100.0)	99.5 (97.0–100.0)
**CSG (capillary blood sample)**^b^	50.0 (11.8–88.2)	62.5 (24.5–91.5)	62.5 (24.5–91.5)	55.6 (21.2–86.3)	90.9 (83.4–95.8)	92.8 (85.7–97.1)	92.8 (85.7–97.1)	92.7 (85.5–97.0)
**CSG (venous blood sample)**^b^	83.3 (35.9–9.6)	87.5 (47.4–88.7)	87.5 (47.4–88.7)	77.8 (40.0–97.2)	98.0 (92.9–99.8)	100.0 (96.3–100.0)	100.0 (96.3–100.0)	100.0 (96.2–100.0)

^a^n = 227

^b^n = 105

**Fig 2:**
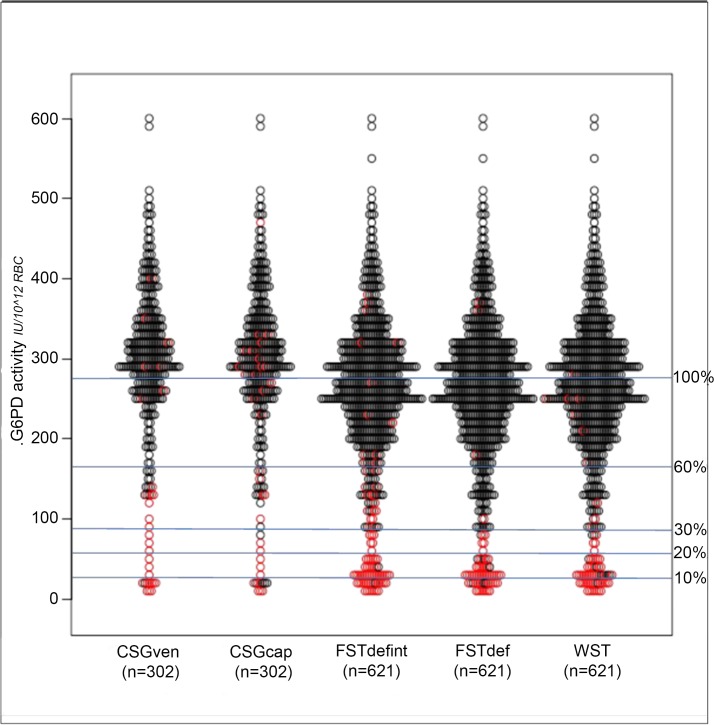
Distribution of the qualitative test results of study participants by G6PD activity Legend: Results are presented per test performed. Red circles indicate a G6PD deficient result, black circles indicate a G6PD normal result, horizontal lines indicate G6PD activity at 10%, 20%, 30%, 60% and 100% G6PD activity. CSGven = Carestart G6PD RDT from venous sample, CSGcap = Carestart G6PD RDT from capillary sample, FSTdefint = Fluorescent spot test considering intermediate results as G6PD deficient, FSTdef = Fluorescent spot test considering intermediate results as G6PD normal, WST = WST 8/1 PMS methoxy test

Table 4 shows the qualitative tests’ results against proportions of the median G6PD activity among male students. We found one FST normal result with a G6PD activity below 10% and two samples with an intermediate FST results below the same cut-off (Table 4 and Fig 2). One of the samples with intermediate FST result and G6PD activity equal to or below 10% was identified as G6PD normal by all other test assays as well. No further overlap in false normal results was detected. The WST returned four G6PD normal results at the same cut-off activity, while the CSG performed on capillary blood returned 4 false normal results and the same test performed on venous blood returned a single false normal result only (Table 4).

**Table 4:** Test results by percent G6PD activity of adjusted median for male students.

Test	Test result	G6PD activity				
		≤10%	≤20%	≤30%	≤60%	>60%
**Fluorescent Spot Test**	Normal	1	2	2	35	504
	Intermediate	2	4	5	21	7
	Deficient	33	46	50	51	3
**Colorimetric Test (WST)**	Normal	4	7	9	55	508
	Deficient	32	45	48	52	6
**CSG (capillary blood sample)**	Normal	4	4	5	18	252
	Deficient	7	10	11	16	16
**CSG (venous blood sample)**	Normal	1	1	1	12	261
	Deficient	10	13	15	22	7

### Operational Characteristics

Both the FST and the WST require a water bath; test interpretation of the FST requires a UV lamp. The CSG, in contrast, does not require additional equipment. While the CSG can be stored at room temperature, the FST and WST require a refrigerator or freezer. Once reconstituted, the FST substrate solution is stable for two weeks if stored frozen, one week if stored at 2–8°C, and 4 hours at room temperature. There is one product, SQMMR G-6-PD qualitative kit (R&D Diagnostics, Ltd., Greece), that does not require water bath incubation and has a short waiting time of 10 minutes before reading of the blood spot. According to its package insert, however, the test must be carried out at 25°C. The mean temperature in Palawan in 2014 and 2015 ranged from 24.6°C to 31.8°C.

WST reagents must be stored in the dark as some reagents are light-sensitive. According to package insert, the WST can be incubated at any temperature between 25°C to 37°C; however, we did not observe any colour development at 25°C and found brightest colour development at 37°C. We found interpretation of the FST and the WST to be subjective and required intense training and experience. Interpretation of the CSG was simpler but hampered by the absence of a control line (Table 5).

**Table 5:** Operational characteristics of G6PD screening kits.

	Procedure No. 203 (FST; Trinity Biotech, Ireland)	WST-8 Assay (WST; Dojindo, Japan)	Carestart™ (CSG; Access Bio, USA)
Cost per assay	USD 9.0	USD 3.2	USD 1.5
Storage conditions	2–8°C	Minus 20°C	4–30°C
Equipment	UV lamp, water bath, pipettor	Water bath, pipettor	None
Reagents	Shelf life of the substrate solution is 2 weeks maximum	Light sensitive reagent	None
Time to result (minutes)	45*	60*	10
Interpretation of results	Experience required to distinguish between intermediate and normal test result	Difficult to distinguish intermediate from normal test results	No control line; Interpretation of very faint results complicated

*5 minutes for sample preparation assumed

## Discussion

Normal (100%) G6PD activity was defined as 270.5 IU/10^12^ RBC or 9.8 IU/g Hb based on the adjusted male median G6PD activity [13]. In the absence of an internationally accepted threshold for G6PD deficiency, this approach defines G6PD deficiency categories proposed by the WHO [21]. The respective value is a study population specific performance indicator which is not universally applicable. A threshold activity of 30% (of the adjusted male median activity) had earlier been proposed as the cut-off above which primaquine treatment can be considered safe [20]. More than 9% of the study participants had G6PD activities below 30%, a higher percentage than reported from an ongoing Philippines’ newborn screening program [22] but within the range of earlier population-based reports [23, 24]. The observed higher number of female than male participants with intermediate G6PD activities could be heterozygous participants, while the intermediate results of male participants indicate a class III G6PD variant.

Given that the FST is the most widely used assay for the diagnosis of G6PDd in Asia [25–27], any other assay will need to perform at least as well. In order to accommodate for the semi-quantitative outcome of the fluorescent spot test (FST), we considered FST intermediate results as either G6PD deficient or in a secondary analysis as G6PD normal. For the guidance of 8-aminoquinoline treatment, health care providers may wish to choose the safest approach and treat patients with intermediate results as G6PD deficient. When defining FST intermediate results as G6PD deficient and any spectrophotometry based G6PD activity beyond 30% of the adjusted male median as G6PD deficient, the sensitivity of the FST in this study was in the same range as earlier reports [19, 20, 28–30].

In comparison to the FST, the observed sensitivity of the WST was significantly lower at 30% G6PD activity. When evaluating the WST, we performed a manual readout and did not distinguish between intermediate and deficient test results as testing staff were not confident to do so. Test performers were therefore asked to consider any doubtful result as G6PDd. While this may be applicable for scenarios involving radical cure for vivax malaria, this will have had an effect on the observed performance of the test. Kuwahata et al [31] in 2010 had modified the test assay to a 96-well plate format that can be interpreted with a standard ELISA reader and evaluated the protocol on the Solomon Islands. The authors concluded that the new protocol provided a valuable tool for population screening. In 2012, De Niz et al. [32] applied this protocol in a study on 235 Ugandan children and compared the obtained results against a quantitative assay. The authors reported a sensitivity of 72% at a 30% G6PD cut-off activity, a value lower as has been observed by us when applying a manual readout. Despite the lower reported performance of the WST, this approach renders the assay a convenient tool for processing high numbers of samples in short periods, addresses the observed difficulties in test interpretation observed by us and accommodates the kits more demanding storage requirements.

Several studies have been conducted in the recent years on the CSG considering spectrophotometry as the gold standard. In 2010, Kim et al [16] conducted a field evaluation of the CSG in more than 900 Cambodian participants on venous blood and reported a sensitivity of 68%. Three years later, in 2013, a second study [19] in the same area evaluated the next generation of the test on capillary blood and observed a markedly improved sensitivity of 100%. Bancone et al [20] observed a sensitivity of 89.1% at 30% G6PD cut-off activity in tests performed on venous blood from 150 Thai participants and Adu Gyasi et al [33] recently reported a sensitivity of 100% in capillary blood samples of 206 Ghanese participants, however applying a cut-off activity of 75%.

We performed the CSG on capillary and on venous blood samples to assess potential differences in performance. While sensitivity of the CSG when performed on venous blood (CSGven) was comparable to the FSTdefint and earlier reports of the second generation of tests, the test was significantly less sensitive when performed on capillary blood (CSGcap). It had been proposed earlier that there may be hematological differences in hemoglobin, hematocrit and RBC count in between capillary and venous blood samples [20], problematic as samples for spectrophotometry are mostly collected from venous blood, whereas, the CSG in most cases is performed on capillary blood. Bancone et al [20] recently compared capillary and venous blood samples from healthy volunteers. While the authors found significant differences in between all three parameters, these did not have a significant influence on FST and CSG results performed on either sample. In contrast, we found sensitivity, irrespective of cut-off activity, to be lower in all cases when the test was performed on capillary blood. As test interpretation in all cases was performed by the same staff and the observed sensitivities from venous blood samples were well above 90% and comparable to results reported by Bancone et al [20], we do not believe this to be the result of a systematic error but possibly due to variations in RBC count or hematocrit value. [34]

Furthermore, an accompanying color chart is helpful in interpretation of the FST and WST tests but the quality of the chart in package inserts must have added value (i.e., high-quality print) in interpretation of the result, especially if the targeted users are community health workers in rural clinics. However, color charts might not ideally address the interpretation of faint color development in test strips.

## Conclusion

The FST had the highest sensitivity among all three test formats. The CSG, in contrast, is the only test that does not require laboratory infrastructure. The sensitivity of the test, when performed on venous blood, was comparable to the FST, and the number of false G6PD normal results was very low. This supports earlier reports that the CSG provides a fair alternative to the FST considering operational and performance characteristics, and suitable for use in malaria endemic areas in the Philippines where basic laboratory equipment are lacking. There is major drawback, however, with the use of CSG on venous blood. Blood collection for malaria diagnosis by volunteer community health workers in the Philippines is limited to finger prick collection. Our observations underscore the need for improved test kit sensitivity using capillary blood. G6PD deficiency testing is envisioned to be utilized by community health workers in the Philippines who are not allowed to perform venipuncture.
